# Prognostic and clinicopathological value of the controlling nutritional status score in patients with multiple myeloma: a meta-analysis

**DOI:** 10.3389/fonc.2025.1517223

**Published:** 2025-03-18

**Authors:** Yin Jin, Wenfei Gu

**Affiliations:** Clinical Laboratory, Huzhou Central Hospital, Affiliated Central Hospital of Huzhou University, Huzhou, Zhejiang, China

**Keywords:** CONUT score, multiple myeloma, meta-analysis, evidence-based medicine, prognosis

## Abstract

**Background:**

The effect of the controlling nutritional status (CONUT) score on forecasting multiple myeloma (MM) prognosis is previously analyzed, whereas the results remained inconsistent. The present meta-analysis focused on identifying the exact function of CONUT in forecasting MM prognosis.

**Methods:**

Web of Science, PubMed, Embase, CNKI, and Cochrane Library were comprehensively searched between inception and 1 February 2025. The effect of CONUT on forecasting MM overall survival (OS) and progression-free survival (PFS) was determined by computing pooled hazard ratios (HRs) together with 95% confidence intervals (CIs).

**Results:**

There were nine studies with 1,176 patients being recruited into the present work. As indicated by our pooled data, elevated CONUT was related to the dismal OS (HR = 1.87, 95% CI = 1.37–2.54, *p* < 0.001) of patients with MM. Nonetheless, CONUT was not significantly related to PFS (HR = 1.33, 95% CI = 0.81–2.19, *p* = 0.254) of MM. Furthermore, higher CONUT score showed a significant relationship to bone marrow plasma cells >30% (OR = 2.30, 95% CI = 1.32–3.99, *p* = 0.003). On the other hand, CONUT was not markedly correlated with gender (OR = 2.68, 95% CI = 0.81–8.82, *p* = 0.105), ISS stage (OR = 1.28, 95% CI = 0.94–1.75, *p* = 0.119), or ECOG PS (OR = 1.30, 95% CI = 0.84–2.01, *p* = 0.234) of MM.

**Conclusion:**

Collectively, according to our results in this meta-analysis, higher CONUT score is markedly related to dismal OS, but not PFS in patients with MM. CONUT score can be used as a candidate marker used to predict MM prognosis in the clinic in the future.

## Introduction

Multiple myeloma (MM), the plasma cell-malignant cancer accumulating within the bone marrow, can result in bone damage and marrow failure (1). Generally, malignant plasma cells can be detected from the bone marrow, which produce abnormal antibodies (M-protein) (2). MM ranks second among hematological malignancies, occurring in 6.5 people per 100,000 worldwide (3). According to GLOBOCAN, 176,404 newly diagnosed MM cases along with 117,077 death cases were reported in 2020 globally (4). There have been considerable advancements in treatment options for MM in recent decades, including proteasome inhibitors, immunomodulatory drugs, and anti-CD38 antibody immunotherapies (5). Despite these advances, most MM patients eventually relapse and cannot be cured, with 5- and 10-year survival rates of 55.6% and 17%, respectively (6, 7). Prognostic markers are important to improve the survival outcomes from MM (8). Consequently, identifying new and reliable markers for predicting MM prognosis is urgently needed.

Current lines of evidence show that inflammation and nutrition play pivotal roles in cancer progression and development (9). In recent years, many nutrition-related indexes, including prognostic nutritional index (PNI) (10), geriatric nutritional risk index (GNRI) (11), albumin-to-globulin ratio (12), and C-reactive protein-to-albumin ratio (CAR) (13), represent key markers for predicting cancer prognosis. First proposed by Ignacio et al. in 2005, the controlling nutritional status (CONUT) score has been used to detect hospital undernutrition (14). In peripheral blood, cholesterol, albumin, and lymphocyte levels can be easily calculated to determine the CONUT score, which ranges from 0 to 12 (**Table 1**). Many studies have shown the prominent significance of CONUT on predicting cancer prognosis such as pancreatic ductal carcinoma (15), cervical cancer (16), esophageal squamous cell carcinoma (17), colon cancer (18), and non-small cell lung cancer (NSCLC) (19). The impact of CONUT on predicting MM prognosis is analyzed previously, whereas conflicting results are reported (20–28). Consequently, this meta-analysis focused on examining the exact impact of CONUT on forecasting MM prognosis. Additionally, we also investigated the relationship of CONUT with clinicopathological factors of MM through this meta-analysis.

**Table 1:** The scoring system of CONUT.

Parameters	Degree			
	Normal	Mild	Moderate	Severe
Albumin level (g/dl)	3.5–4.5	3.00–3.49	2.50–2.99	<2.50
Score	0	2	4	6
Total lymphocytes/ml	≥1,600	1,200–1,599	800–1,199	<800
Score	0	1	2	3
Cholesterol (mg/dl)	>180	140–180	100–139	<100
Score	0	1	2	3
Screening total score	0–1	2–4	5–8	9–12

## Materials and methods

### Study guidelines

This meta-analysis was carried out following the Preferred Reporting Items for Systematic Reviews and Meta-Analyses (PRISMA) guidelines (29).

### Literature retrieval

PubMed, Web of Science, Embase, Cochrane Library, and CNKI were thoroughly searched between inception and 1 February 2025 using the search items below: (controlling nutritional status score or CONUT or controlling nutritional status) and (multiple myeloma or myeloma). There was no restriction on the language of publications. Reference lists of electronically selected articles were checked manually to obtain more relevant articles.

### Eligibility criteria

Articles were included based on the following criteria: 1) pathological diagnosis of MM was made; 2) the CONUT was determined based on pretreatment peripheral blood test; 3) those that mentioned the association between CONUT and MM survival outcomes; (4) those that provided available or computed hazard ratios (HRs) and 95% confidence intervals (CIs); (5) threshold CONUT was available to stratify the patients; and (6) studies published in any language. The following articles were eliminated: 1) reviews, comments, letters, meeting abstracts, and case reports; 2) publications with duplicated or overlapped data; and 3) animal studies.

### Data collection and quality evaluation

Two authors (YJ and WG) independently collected data from the qualified articles. Any dispute between them was solved through discussion until reaching a consensus. The following data were extracted: first author, year, country, sample size, gender, age, study design, study period, International Staging System (ISS) stage, threshold, threshold determination approach, follow-up, survival endpoints, and survival analysis types, as well as HRs with 95% CIs. We selected overall survival (OS) and progression-free survival (PFS) as our primary and secondary survival outcomes, respectively. Newcastle-Ottawa Scale (NOS) scores were used to evaluate study quality (30), with studies scoring 6 or higher considered high quality.

### Statistical analysis

The effect of CONUT on forecasting MM OS and PFS was analyzed through calculating pooled HRs and 95% CIs. Among-study heterogeneities were assessed through Cochran’s *Q*-test and *I* ^2^ statistics. *I* ^2^ >50% and *p <*0.10 (*Q*-test) indicate obvious heterogeneity, so the random-effects model is chosen; otherwise, the fixed-effects model is used. Meanwhile, the prognostic value of CONUT in various patient subgroups was investigated through subgroup analysis. Also, the heterogeneity source was identified, and the pooled result stability was evaluated by conducting sensitivity analysis. The relationship of CONUT with MM clinicopathological characteristics was assessed based on pooled odds ratios (ORs) and 95% CIs. Begg’s test and Egger’s test were adopted in evaluating publication bias. Stata version 12 (StataCorp, College Station, TX, USA) was applied in the statistical analyses. A *p*-value <0.05 indicated statistical significance.

## Results

### Literature search process

**Figure 1:**
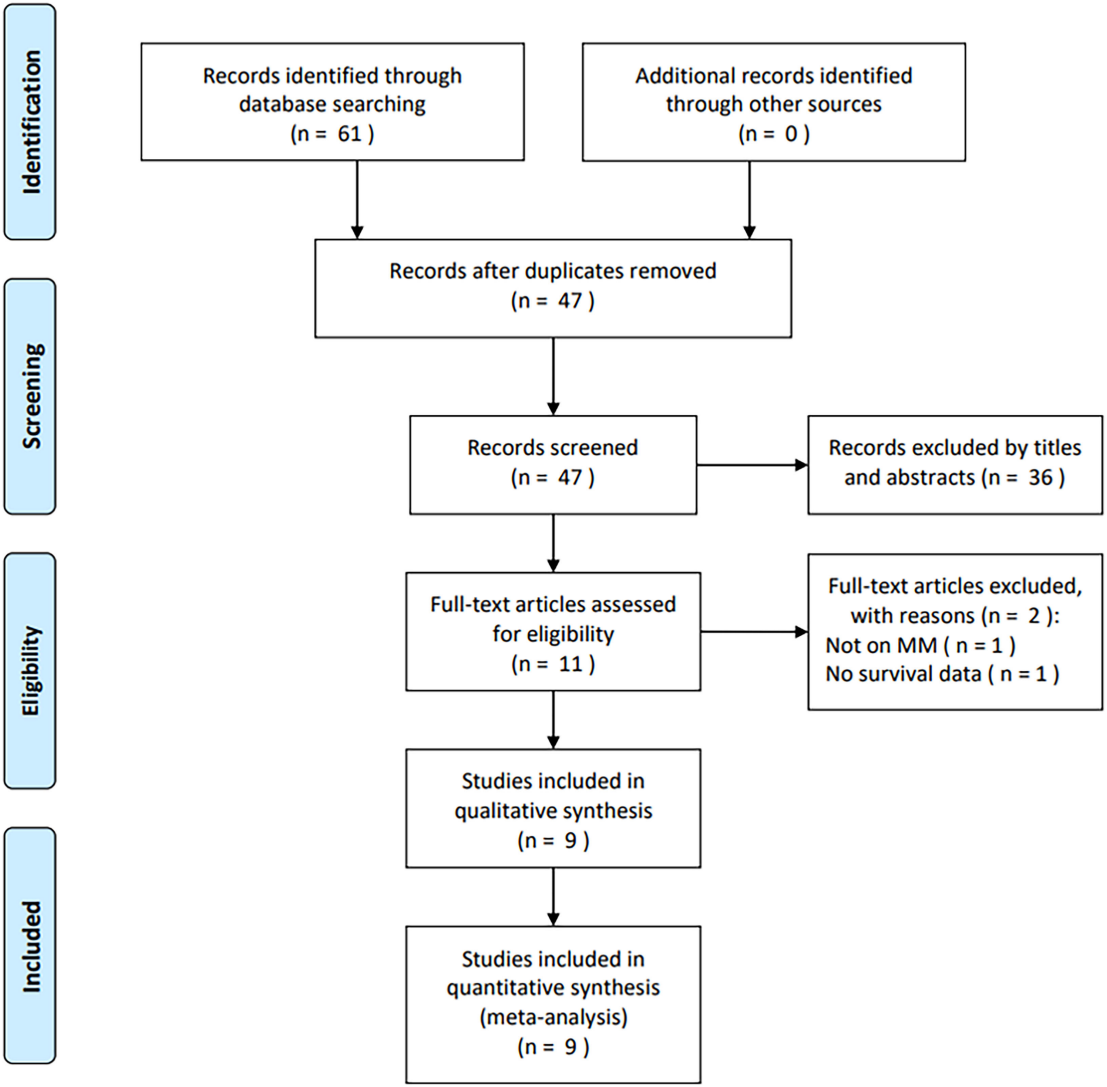
PRISMA flowchart in this meta-analysis.

Through primary search, a total of 61 studies were obtained, with 47 being maintained when removing duplicates (**Figure 1**). Through title and abstract checking, 36 studies were excluded because of irrelevance and animal studies. Subsequently, the full texts of 11 studies were assessed, with two being eliminated due to not focusing on MM (*n* = 1) and no survival data provided (*n* = 1). Ultimately, nine studies with 1,176 patients (20–28) were enrolled for the present meta-analysis (**Figure 1**).

### Identified study features

**Table 2** displays the identified study features. The publication year was 2020–2024 (20–28). Two studies were conducted in Japan (20, 21) and seven in China (22–28). Three studies were published in English (20, 21, 24) and six were in Chinese (22, 23, 25–28). Their sample sizes were 57–245 (median, 119). The enrolled studies were of retrospective design. Eight studies recruited ISS stage I–III patients (20–26, 28) and one enrolled patient with recurrent disease (27). In terms of cutoff value, three studies adopted ≥3.5 (22, 23, 27), three used ≥5 (20, 21, 28), two selected ≥6.5 (25, 26), and one used ≥3 (24). Six studies determined the cutoff value by using the receiver operating characteristic (ROC) curve (20, 22, 23, 25–27), two studies referred to the literature (21, 28), and one adopted the X-tile software (24). All nine studies mentioned the relationship of CONUT with OS (20–28) and three studies showed the significance of CONUT in predicting PFS (22, 26, 28) in MM. Five articles obtained the HRs and 95% CIs from univariate regression (22, 25–28) and four studies adopted multivariate analysis (20, 21, 23, 24). The NOS scores were 7–9, suggesting high quality (**Table 2**).

**Table 2:** Baseline characteristics of the included studies in this meta-analysis.

Study	Year	Country	Sample size	Gender (M/F)	Age (years) Median (range)	Study design	Study period	ISS stage	Cutoff value	Cutoff determination	Follow-up (months) Median (range)	Survival outcomes	Survival analysis	NOS score
Kamiya, T (20).	2020	Japan	178	85/93	69 (39–86)	Retrospective	2005–2018	I–III	≥5	ROC curve	1–140	OS	Multivariate	8
Okamoto, S (21).	2020	Japan	64	33/31	66 (41–84)	Retrospective	2008–2017	I–III	≥5	Literature	1–120	OS	Multivariate	7
Li, Y. Q (22).	2021	China	119	78/41	56 (23–83)	Retrospective	2010–2018	I–III	≥3.5	ROC curve	1–144	OS, PFS	Univariate	8
Liang, F (23).	2021	China	157	88/69	64 (30–91)	Retrospective	2014–2018	I–III	≥3.5	ROC curve	24 (0.5–71.0)	OS	Multivariate	8
Zhou, X (24).	2021	China	245	145/100	65 (33–83)	Retrospective	2007–2017	I–III	≥3	X-tile	38 (1–154)	OS	Multivariate	9
Ma, K. W (25).	2023	China	203	110/93	64 (24–83)	Retrospective	2007–2019	I–III	≥6.5	ROC curve	1–150	OS	Univariate	8
Xu, X. Z (26).	2023	China	57	34/23	54 (27–72)	Retrospective	2016–2018	I–III	≥6.5	ROC curve	43.8 (0.7–78.1)	OS, PFS	Univariate	7
Li, Q. F (27).	2024	China	74	46/28	68 (65–80)	Retrospective	2020–2022	Recurrent	≥3.5	ROC curve	7 (1–17)	OS	Univariate	7
Xiong, Y. Y (28).	2024	China	79	36/43	54 (33–67)	Retrospective	2011–2020	I–III	≥5	Literature	33.4 (4.4–124.1)	OS, PFS	Univariate	8

OS, overall survival; PFS, progression-free survival; ROC, receiver operating characteristic; NOS, Newcastle-Ottawa Scale; M, male; F, female; ISS, International Staging System.

### CONUT and OS

**Figure 2:**
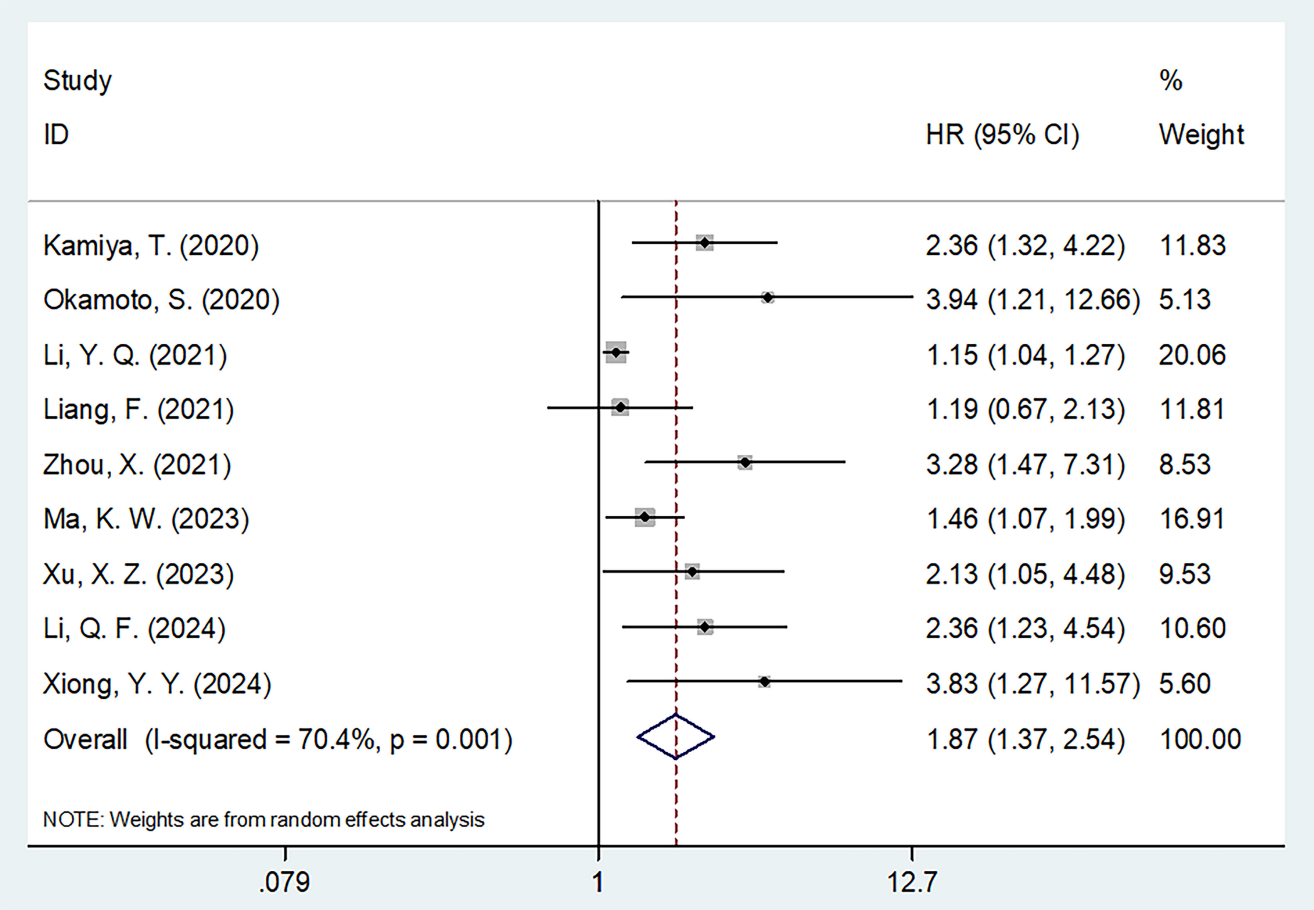
Forest plots of the association between CONUT score and OS in patients with multiple myeloma.

All nine studies (20–28) provided the information regarding the impact of CONUT on forecasting OS of MM patients. Due to significant heterogeneity (*I* ^2^ = 70.4%, *p* = 0.001), this study adopted the random-effects model. As indicated by our combined data, higher CONUT was connected with unfavorable OS of MM patients (HR = 1.87, 95% CI = 1.37–2.54, *p* < 0.001; **Table 3**, **Figure 2**). As shown by subgroup analysis, high CONUT remained the significant prognostic indicator of dismal OS, despite country, sample size, stage, threshold determination, or survival analysis (all *p* < 0.05; **Table 3**).

**Table 3:** Subgroup analysis of the prognostic value of CONUT for OS and PFS in patients with MM.

Subgroups	No. of studies	No. of patients	Effects model	HR (95% CI)	*P*	Heterogeneity *I* ^2^ (%) Ph	
OS							
Total	9	1,176	Random	1.87 (1.37–2.54)	<0.001	70.4	0.001
Country							
Japan	2	242	Fixed	2.61 (1.55–4.39)	<0.001	0	0.445
China	7	934	Random	1.69 (1.24–2.30)	<0.001	67.8	0.005
Sample size							
<100	4	274	Fixed	2.61 (1.72–3.95)	<0.001	0	0.723
≥100	5	902	Random	1.54 (1.12–2.11)	0.008	70.1	0.010
ISS stage							
I–III	8	1,102	Random	1.81 (1.31–2.49)	<0.001	70.1	0.001
Recurrent	1	74	–	2.36 (1.23–4.54)	0.010	–	–
Cutoff value							
≥3	1	245	–	3.28 (1.47–7.31)	0.004	–	–
≥3.5	3	350	Random	1.35 (0.93–1.97)	0.119	55.9	0.103
≥5	3	321	Fixed	2.80 (1.75–4.48)	<0.001	0	0.618
≥6.5	2	260	Fixed	1.54 (1.16–2.06)	0.003	0	0.346
Cutoff determination							
Literature	2	143	Fixed	3.88 (1.74–8.67)	0.001	0	0.974
ROC curve	6	788	Random	1.54 (1.17–2.03)	0.002	63.6	0.017
X-tile	1	245	–	3.28 (1.47–7.31)	0.004	–	–
Survival analysis							
Univariate	5	532	Random	1.64 (1.16–2.30)	0.005	68.8	0.012
Multivariate	4	644	Random	2.21 (1.31–3.74)	0.003	50.7	0.107
PFS							
Total	3	255	Random	1.33 (0.81–2.19)	0.254	55.5	0.106
Sample size							
<100	2	136	Random	1.60 (0.66–3.84)	0.295	70.5	0.066
≥100	1	119	–	1.08 (0.71–1.62)	0.727	–	–
Cutoff determination							
Literature	1	79	–	2.56 (1.23–5.35)	0.012	–	–
ROC curve	2	176	Fixed	1.07 (0.76–1.50)	0.711	0	0.940

CONUT, controlling nutritional status; OS, overall survival; PFS, progression-free survival; MM, multiple myeloma; ROC, receiver operating characteristic; ISS, International Staging System.

### CONUT and PFS

**Figure 3:**
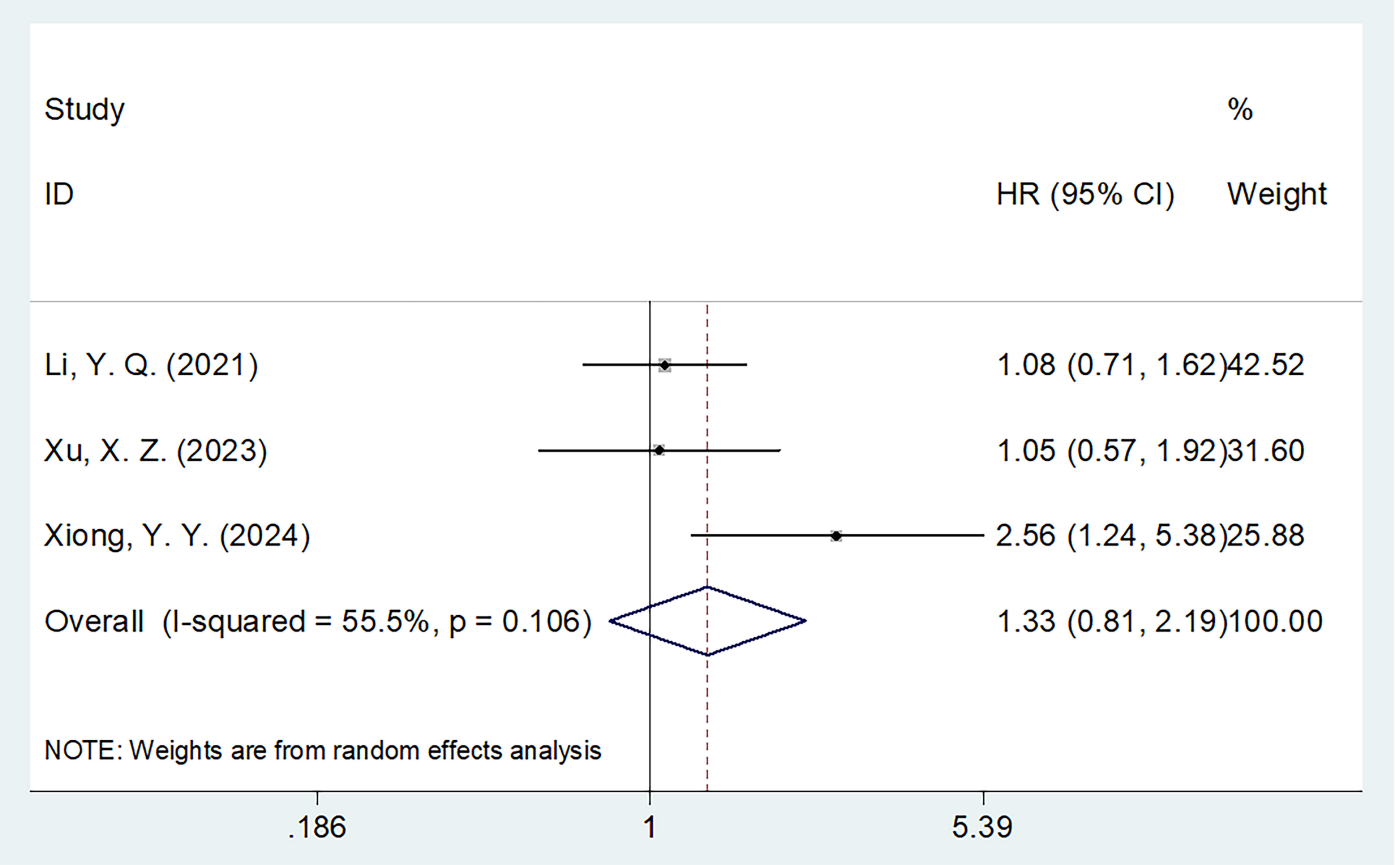
Forest plots of the association between CONUT score and PFS in patients with multiple myeloma.

Three studies consisting of 255 patients (22, 26, 28) reported the correlation between CONUT and PFS in MM. The combined results suggested HR = 1.33, 95% CI = 0.81–2.19, *p* = 0.254, indicating that CONUT was not a significant prognostic marker for PFS in MM (**Table 3** **;** **Figure 3**). Subgroup analysis demonstrated that elevated CONUT exhibited obvious relationship to PFS in MM using the threshold determined according to the literature (*p* = 0.012; **Table 3**).

### The relationship of CONUT with MM clinicopathological factors

**Figure 4:**
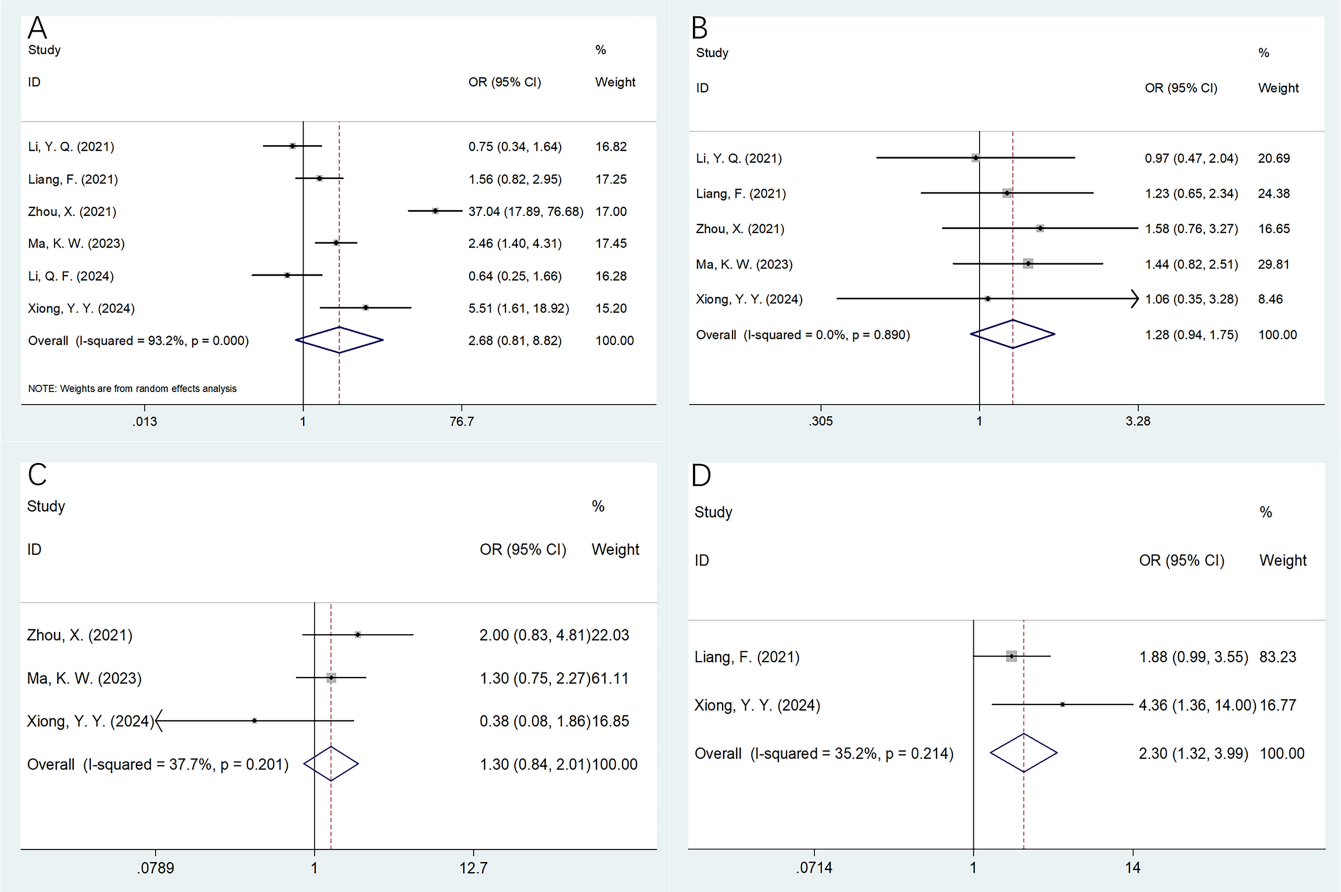
Forest plots of the correlation between CONUT and clinicopathological features of multiple myeloma. **(A)** Gender (male vs. female); **(B)** ISS stage (III vs. I–II); **(C)** ECOG PS (≥2 vs. <2); and **(D)** bone marrow plasma cells (>30% vs. ≤30%).

A total of six studies including 877 cases (22–25, 27, 28) presented the relationship of CONUT with MM clinicopathological factors. Based on our combined findings, higher CONUT score showed a significant relationship to bone marrow plasma cells >30% (OR = 2.30, 95% CI = 1.32–3.99, *p* = 0.003; **Table 4**, **Figure 4**). Nonetheless, CONUT was not apparently connected with gender (OR = 2.68, 95% CI = 0.81–8.82, *p* = 0.105), ISS stage (OR = 1.28, 95% CI = 0.94–1.75, *p* = 0.119), or Eastern Cooperative Oncology Group Performance Status (ECOG PS) (OR = 1.30, 95% CI = 0.84–2.01, *p* = 0.234) of MM (**Table 4**, **Figure 4**).

**Table 4:** The association between CONUT and clinicopathological features in patients with MM.

Clinicopathological features	No. of studies	No. of patients	Effects model	OR (95% CI)	*P*	Heterogeneity *I* ^2^ (%) Ph	
Gender (male vs. female)	6	877	Random	2.68 (0.81–8.82)	0.105	93.2	<0.001
ISS stage (III vs I–II)	5	803	Fixed	1.28 (0.94–1.75)	0.119	0	0.890
ECOG PS (≥2 vs. <2)	3	527	Fixed	1.30 (0.84–2.01)	0.234	37.7	0.201
Bone marrow plasma cells (>30% vs. ≤30%)	2	236	Fixed	2.30 (1.32–3.99)	0.003	35.2	0.214

CONUT, controlling nutritional status; MM, multiple myeloma; ISS, International Staging System; ECOG PS, Eastern Cooperative Oncology Group Performance Status.

### Sensitivity analysis

**Figure 5:**
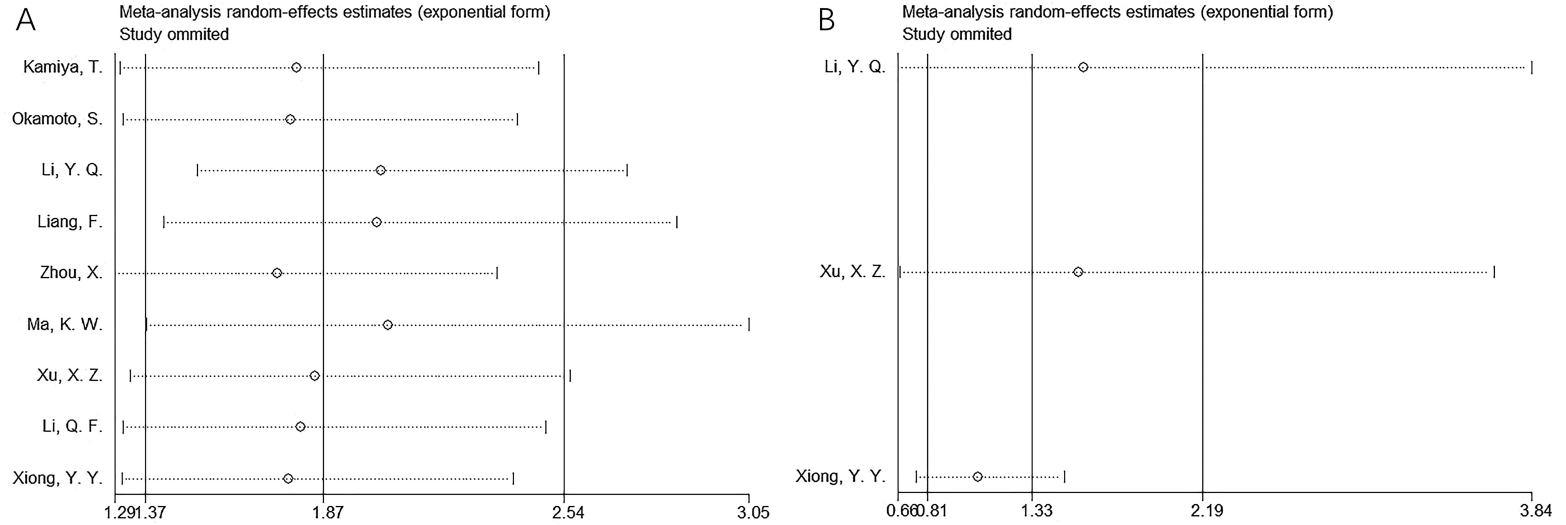
Sensitivity analysis. **(A)** OS and **(B)** PFS.

Sensitivity analysis indicated that no single study significantly impacted the effect size for the relationship between CONUT and OS or PFS of MM (**Figure 5**), which suggested that our results were reliable.

### Publication bias

**Figure 6:**
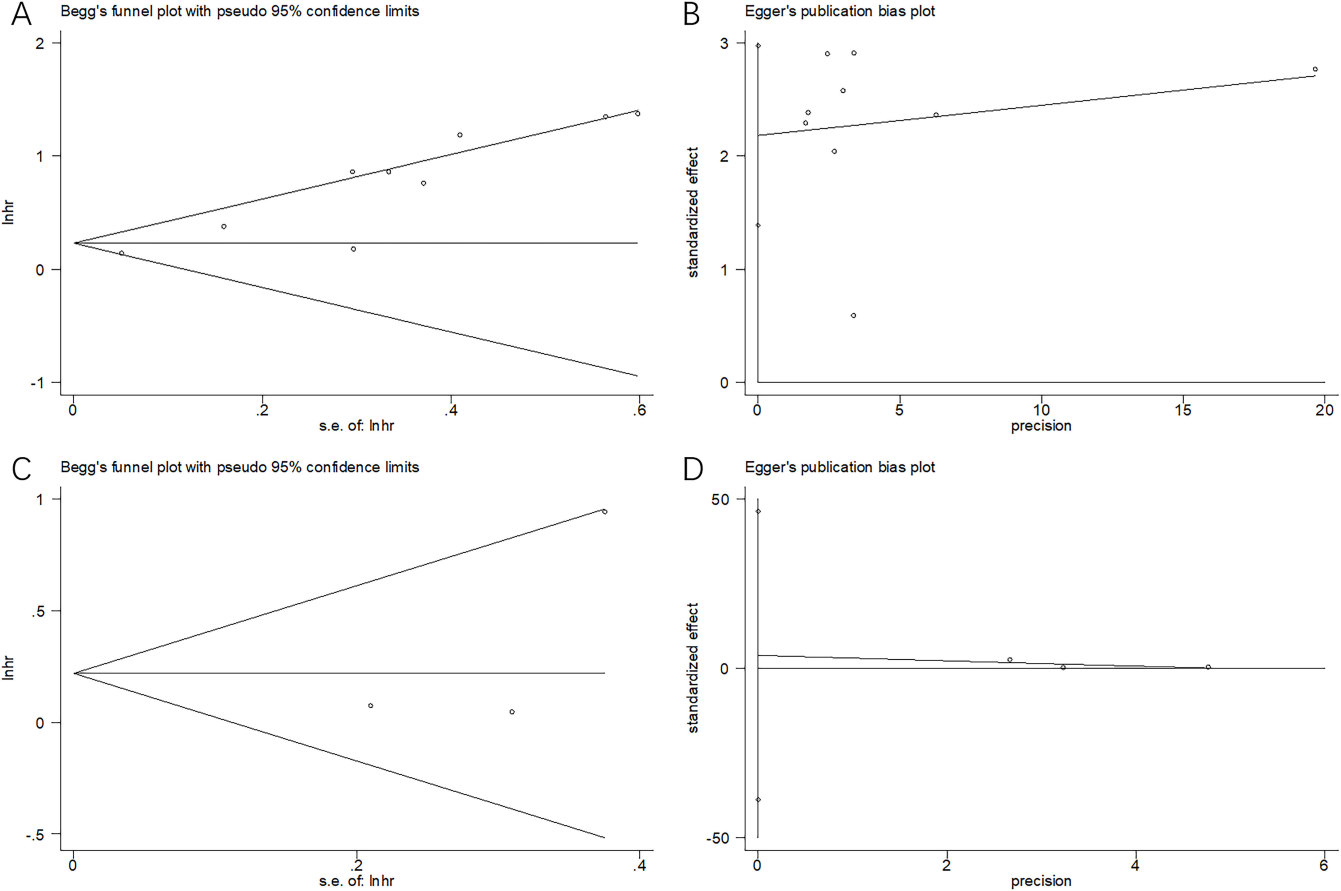
Publication bias test. **(A)** Begg’s test for OS, *p* = 0.118; **(B)** Egger’s test for OS, *p* = 0.102; **(C)** Begg’s test for PFS, *p* = 0.296; and **(D)** Egger’s test for PFS, *p* = 0.462.

This study utilized Begg’s and Egger’s tests in examining potential publication bias, which existed in the present work (*p* = 0.118 and 0.102 for OS; *p* = 0.296 and 0.462 for PFS through Begg’s and Egger’s tests, respectively) (**Figure 6**).

## Discussion

The value of CONUT in forecasting MM prognosis is widely explored, with controversial results presented. This work aggregated data in nine articles with 1,176 patients to shed light on this issue. As discovered, higher CONUT significantly forecasted the dismal OS of MM. Nonetheless, CONUT was not significantly related to PFS in patients with MM. Furthermore, according to our meta-analysis, higher CONUT showed an obvious relationship to bone marrow plasma cells >30% in MM. Our results were robust, as validated by sensitivity and publication bias analyses. Higher CONUT was a significant prognostic factor for OS, but not PFS in MM. For the first time, this meta-analysis investigated the effect of CONUT on predicting MM prognosis.

CONUT is calculated by serum albumin, cholesterol, and total lymphocyte count, and reductions of these three elements lead to high CONUT. The mechanisms related to the effect of CONUT on predicting OS in MM remain to be fully illustrated, yet they may be explained below. Firstly, a patient’s serum albumin, the most common plasma protein, can serve as an objective measure of their nutritional status (31). Albumin directly indicates a host’s nutritional status, and it performs an essential function, such as damage repair and material transport, during pathophysiological processes (32). Lower serum albumin contents are indicative of chronic inflammation activation and low nutritional status. Secondly, as a key component in the human immune system, lymphocytes suppress carcinogenesis and recurrence by producing cytokines and inducing cytotoxic death, thereby regulating immune function (33). The lymphocytes are also critical to maintaining the adaptive immune system, and a decreased lymphocyte count is associated with dismal prognostic outcome of many cancers (34). It is common for lymphocytopenia to be accompanied by an increase in leukocytes, which may allow tumor cells to hide from the immune system (35). Thirdly, in healthy cells, cholesterol is a key structural component of lipid metabolism, and it impacts the membrane properties like function and fluidity of membrane proteins (36). The presence of cholesterol on the cellular membrane is closely related to tumor proliferation. Moreover, patients having lower cholesterol levels usually do not show the best prognostic outcome (37). Cancer development and tumorigenesis are linked to cholesterol metabolism disorders. Consequently, a reduction of serum cholesterol suggests the reduction of cell membrane cholesterol, which affects the cancer-fighting capacity of immune-active cells.

Notably, this meta-analysis revealed that CONUT was a significant prognostic marker for patients with MM, which could be considered an effective biomarker in clinical practice. Current lines of evidence have shown that there are many types of prognostic markers for MM, such as serum free light chain (FLC) levels (38), generation sequencing (NGS) panels (39), circulating tumor cells (CTCs) (40), proteomics panels (41), and genomic biomarkers (8, 42). Compared with these prognostic markers, CONUT score has the following strengths. First, CONUT is easily available. CONUT is derived from blood test results, being commonly examined in clinical management. Second, the prognostic efficiency of CONUT is reliable. The current meta-analysis indicated that CONUT score is a significant prognostic biomarker for long-term survival outcomes in MM. Third, the CONUT test is cost-effective and did not increase medical expenses because CONUT can be calculated from blood tests and no specific examination for CONUT is needed. Therefore, CONUT could serve as a promising and cost-effective prognostic marker for MM in clinical settings.

Recently, many articles also reported the effect of CONUT on predicting cancer prognosis through meta-analysis (43–47). Wang et al. showed that higher CONUT score showed a close relationship with OS and disease-free survival (DFS) in head and neck cancer through their meta-analysis including 1,478 cases (43). As reported by Li et al., increased CONUT scores showed a close relationship to the dismal OS and PFS of lymphoma in their meta-analysis involving seven studies (44). As demonstrated by one latest meta-analysis comprising 17 articles, a high CONUT score increased the risk of tumor progression, advanced tumor stage, microvascular invasion, and postoperative complications in gastric cancer patients (45). Niu et al. discovered from a meta-analysis involving 2,569 cases that increased CONUT scores had a close relationship to the reduced OS and PFS of gynecological cancer (46). According to Peng et al. in a meta-analysis including 3,029 cases, a high CONUT score was positively related to dismal prognoses in NSCLC (47). Our results conform to the prognostic value of CONUT in additional cancers.

This work has some limitations. Firstly, our studies were from Asia, mainly China. Therefore, the results were more applicable to Asian MM patients. Secondly, our sample was limited in size. Although nine studies were included, the total sample size was 1,176, which was relatively small. Thirdly, all included studies were retrospective, which might introduce selection bias. Consequently, large-scale multicenter prospective studies should be conducted for further validation.

## Conclusions

In summary, a higher CONUT score is apparently related to shortened OS but not PFS in patients with MM. CONUT score can be a candidate marker to predict MM prognosis clinically.

## Data Availability

The original contributions presented in the study are included in the article/supplementary material. Further inquiries can be directed to the corresponding author.
